# Investigating Brain Functional Connectivity and Its Correlation With Cognitive Dysfunction in Chronic Kidney Disease Patients via Resting‐State fMRI

**DOI:** 10.1002/brb3.70947

**Published:** 2025-10-10

**Authors:** Ying Liu, Yingying Wang, Liling Peng, Huan Yu, Ning Wu, Chunhua Song, Chaoyang Zhang, Yan Cai, Zhenwei Wang, Yiqing Sun, Xin Gao

**Affiliations:** ^1^ Department of Nephrology Department of Radiology Liangxiang Hospital Beijing China; ^2^ Radiology Center People's Hospital of Xinjiang Uygur Autonomous Region Xinjiang China; ^3^ Shanghai Universal Medical Imaging Diagnostic Center Shanghai China; ^4^ Department of Medical Imaging Yanjing Medical College Capital Medical University Beijing China; ^5^ Department of Radiology Liangxiang Hospital Beijing China; ^6^ Department of Nephrology Beijing BOE Hospital Beijing China; ^7^ Department of Nephrology The Affiliated Hospital of Yangzhou University Yangzhou Jiangsu China

**Keywords:** brain connection, CKD patients, cognitive function, dialysis, fMRI, functional network

## Abstract

**Objective:**

This study aimed to assess the brain functional connectivity and its association with cognitive function in patients with chronic kidney disease (CKD) using resting‐state functional magnetic resonance imaging (rs‐fMRI).

**Methods:**

A total of 64 CKD patients were enrolled and divided into two groups based on their dependence on dialysis: dialysis‐dependent CKD (DD‐CKD) group (*n* = 38) and non‐dialysis‐dependent CKD (NDD‐CKD) group (*n* = 26). A total of 43 healthy controls (NC) were also recruited and matched for age and sex. Cognitive function was evaluated using the Mini‐Mental State Examination (MMSE) and Montreal Cognitive Assessment (MoCA). MRI scans were conducted on a 3.0T Magnetom Skyra scanner equipped with a 32‐channel phased array head coil. Data analysis was performed using the Data Processing Assistant for Resting‐State fMRI (DPARSF) and Statistical Parametric Mapping (SPM) software.

**Results:**

Cognitive scores (MMSE and MoCA) were significantly lower in both CKD groups compared to healthy controls (*p* < 0.001), with DD‐CKD patients exhibiting worse cognitive performance than NDD‐CKD patients (*p* < 0.05). Laboratory parameters also differed: compared with DD‐CKD, NDD‐CKD patients had significantly lower levels of protein, creatinine, calcium, and phosphate (all *p* < 0.05). Network‐based statistical analysis revealed reduced functional connectivity in both CKD groups relative to controls (*p* < 0.05). NDD‐CKD patients showed disruptions mainly in the frontal‐insular and occipital networks, whereas DD‐CKD patients exhibited more extensive alterations involving frontoparietal, cingulate, and visual regions. Correlation analysis further showed that connectivity reductions in key regions—including the dorsolateral prefrontal cortex and parietal association areas—were negatively associated with renal function indicators such as serum creatinine and urea nitrogen (*p* < 0.05).

**Conclusion:**

Resting‐state fMRI effectively reflects alterations in brain functional connectivity in CKD patients and is associated with cognitive performance. Notably, DD‐CKD patients showed more extensive network disruptions and more severe cognitive impairment.

## Graphical abstract

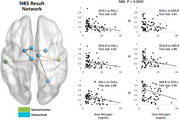

## 1 Introduction

Chronic kidney disease (CKD) arises from the impairment of renal structure due to a variety of causes. Clinical manifestations and pathological indicators of CKD include abnormal glomerular filtration rates (GFRs), anomalies in urine, alterations in blood composition, and imaging abnormalities (Zhang et al. 2022). CKD is frequently accompanied by severe cognitive impairment, with studies indicating that the incidence of cognitive dysfunction may reach as high as 70% in patients with chronic renal failure. The anterior cingulate cortex, a high‐level structure involved in adjusting the brain's connectivity network, undergoes alterations in CKD patients with cognitive impairment (Gallagher 2021; Matsuda 2016). Understanding the neuropathophysiological mechanisms underlying cognitive impairment in CKD patients is essential, as it may enable early clinical intervention to improve both quality of life and long‐term outcomes (Pierce 2011; Maximo et al. 2021). Although the mechanisms remain unclear, growing evidence suggests that central nervous system (CNS) damage in CKD involves a complex interplay of vascular injury, metabolic imbalance, and oxidative stress. Antioxidant systems may offer neuroprotective effects, potentially mitigating CKD‐related cognitive decline (Nazzi et al. 2024).

The exact mechanisms responsible for CNS damage in CKD patients remain elusive. Exploring the relationship between neurological disease and cognitive dysfunction in CKD patients through neuroimaging can provide an objective imaging tool for the prognosis and treatment of CKD patients (Crane et al. 2020). Magnetic resonance imaging (MRI) offers comprehensive spatial and temporal resolution of the entire brain. Resting‐state functional MRI (rs‐fMRI), in particular, is instrumental in probing the connectivity of brain networks. As highlighted by recent theoretical frameworks, including the “self‐studying brain” concept, understanding how the brain monitors and reorganizes itself in both health and disease remains a frontier in cognitive neuroscience (Battaglia et al. 2025). Studies suggest that patients with end‐stage renal disease (ESRD) exhibit anomalies in the structure, function, and metabolism of the anterior cingulate cortex (Li et al. 2022; Zhang et al. 2020; Li et al. 2021).

Previous neuroimaging studies have demonstrated significant abnormalities in brain functional connectivity (FC) among patients with CKD (Qiu et al. 2014; Ni et al. 2014; Huang et al. 2021; Shi et al. 2019; Ma et al. 2020). Notably, patients with diabetic dialysis‐dependent CKD (DD‐CKD) and diabetic non‐dialysis‐dependent CKD (NDD‐CKD) might exhibit distinct patterns of FC alterations due to differences in disease severity, uremic toxin accumulation, and dialysis treatment itself. This study aims to explore the correlation between alterations in brain connectivity functional networks and cognitive dysfunction among dialysis and non‐dialysis CKD patients as well as healthy controls using fMRI. Clarifying these connectivity differences through fMRI could provide valuable neuroimaging markers for better distinguishing cognitive profiles between dialysis and non‐dialysis CKD populations, contributing to more tailored clinical management strategies.

## 2 Material and Methods

### 2.1 Participants

The current investigation was granted ethical clearance by the institutional review board of Beijing Liangxiang Hospital. The study period encompassed the duration from January 2022 to June 2022, during which a total of 64 individuals with CKD were recruited. This cohort was subdivided into 38 patients with DD‐CKD and 26 patients with NDD‐CKD. The enrollment process was conducted in accordance with the ethical principles enshrined in the Declaration of Helsinki and its subsequent amendments, ensuring the responsible handling of biological materials. A control group consisting of 43 healthy participants, matched for age and gender, was also assembled for comparison purposes. The DD‐CKD group comprised 18 males and 20 females, spanning an age range from 23 to 65 years (mean age: 58.06 ± 11.07), whereas the NDD‐CKD group consisted of 17 males and 9 females, aged between 29 and 77 years (mean age: 56.88 ± 11.70). The control group included 28 males and 15 females, covering an age bracket from 18 to 78 years (mean age: 55.64 ± 9.70).

### 2.2 Inclusion Criteria

Participants were diagnosed with CKD and met the diagnostic benchmarks established by the National Kidney Foundation Association's NKF‐KDOQI guidelines. Participants were aged 18 years or older, undergoing chronic hemodialysis or ambulatory peritoneal dialysis for more than 3 months, and free of infections and other complications within the past 3 months. They had a comprehensive medical history and no known contraindications to MRI imaging.

### 2.3 Exclusion Criteria

Individuals with severe neurological impairment within the preceding 6 months, presence of hepatitis, tumors, brain tumors, trauma, epilepsy, or other preexisting medical conditions, major depressive disorder, bipolar disorder, or schizophrenia, and a history of substance abuse or drug dependency were excluded.

### 2.4 Methodology

#### 2.4.1 Cognitive Assessment

All participants underwent a comprehensive cognitive evaluation using the Mini‐Mental State Examination (MMSE) and the Montreal Cognitive Assessment (MoCA). Demographic information and clinical data such as disease duration, dialysis duration, and laboratory examination outcomes were also collected (Viol et al. 2019).

#### 2.4.2 Image Acquisition

The patient was subjected to an MRI scan employing a 32‐channel stage array head coil on a Siemens Germany‐manufactured Magnetom Skyra 3.0T MRI scanner. The cranial MRI was conducted in the supine head‐first position.

1. 3D T1‐weighted structural imaging scan was executed using the MP‐RAGE sequence, employing the following parameters: a repetition time (TR) of 2000 ms, an inversion time (TI) of 880 ms, an echo time (TE) of 2.01 ms, a flip angle (FA) of 8°, a matrix size of 256 × 256, a field of view (FOV) of 256 × 256 mm^2^, a total sagittal thickness of 208 mm, and a slice thickness of 1 mm.
2. The fMRI scan employed a single‐shot gradient echo‐echo planar imaging (SS‐GRE‐EPI) sequence, with a TR of 2000 ms and a TE of 30 ms. The FA was set to 90°, and the matrix size was 64 × 64, with a FOV of 224 × 224 mm^2^. The slice thickness was 4 mm, and the scan comprised 36 slices. In addition, there were 180 repetitions, fat suppression was applied, and a parallel imaging factor of 2 was utilized.

During the resting‐state scan, participants were instructed to close their eyes, lie down, relax, remain awake, and avoid engaging in specific thoughts.

## 3 Data Analysis and Statistics

### 3.1 The rs‐fMRI Data Analysis

The processing of the fMRI data was conducted utilizing the Configurable Pipeline for the Analysis of Connectomes (C‐PAC), which is a Python‐based pipeline tool that integrates AFNI, ANTs, FSL, and bespoke Python scripts. The functional preprocessing phase encompassed the following sequential steps:

1. The elimination of the initial 10 time points;
2. The correction of slice timing;
3. The removal of oblique orientation in the images;
4. The reorientation of images to the right‐to‐left, back‐to‐front, and bottom‐to‐top orientations;
5. The application of motion correction to facilitate the derivation of motion parameters by averaging images;
6. Cranial stripping to remove non‐brain tissue;
7. Normalization of the global mean intensity to a standard value of 10,000;
8. The registration of the functional image to anatomical space, involving linear transformations, transformations based on white matter boundary definitions, and the utilization of prior white matter tissue segmentation from FSL;
9. The utilization of ICA‐AROMA and partial component regression to mitigate motion‐induced artifacts;
10. The application of noise signal regression, which included the averaging of white matter and cerebrospinal fluid signals from prior tissue segmentation, the transformation from anatomical to functional space, the inclusion of motion parameters, linear trends, and global signals.

Following the completion of preprocessing, the brain was segmented into 116 distinct regions of interest (ROIs) employing the AAL Atlas. Thereafter, the mean time series for each ROI was individually extracted to construct the functional brain network. The FC edges were delineated by calculating the Pearson correlation coefficient between every pair of ROIs, resulting in a comprehensive matrix of 116 × 116 edges.

### 3.2 Network‐Based Statistics

To evaluate the statistical significance of disparities in FC between distinct groups, we adopted the network‐based statistics (NBS) methodology. Prior to the application of the NBS statistical framework, *Z*‐scores were adjusted for potential confounders, such as age and gender, through the implementation of linear regression. Subsequently, the NBS approach, utilizing *Z*‐scores, employed an initial statistical threshold to delineate a collection of suprathreshold connections based on the *t*‐statistic.

The significance of each individual component was determined by meticulously examining all connectivity components that fell within the suprathreshold range and recording their respective sizes—denoted by the number of connections. This assessment of significance was derived from the null distribution of the largest possible component size, which was obtained via a non‐parametric permutation technique encompassing 5000 permutations. In the context of assessing group discrepancies in FC, we utilized the NBS tool, an instrument developed by Zalensky. Before the application of the NBS statistical methodology, we adjusted the *Z*‐scores to account for potential confounding variables, including age and gender, through linear regression analysis.

### 3.3 Data Statistics

Cognitive function scores were computed for each subject using Microsoft Excel 2007, and these scores were subsequently reanalyzed within SPSS for Windows version 17.0 (SPSS Inc., Chicago, Illinois, USA). Univariate linear regression analysis was employed to examine the correlation between the total cognitive function score and the scores for Verbal Comprehension Index (VCI), Working Memory Index (WMI), Perceptual Reasoning Index (PRI), Processing Speed Index (PSI), and MMSE, serving as dependent variables. Bilateral outcomes were reported, with statistical significance defined as *p* < 0.05.

In the context of FC statistics utilizing the NBS approach, General Linear Model (GLM) method was employed to assess the discrepancy in FC between patients with CKD and the control group, without adjusting with other parameters. In addition, GLM was used to investigate the correlation between FC and kidney function. The results were considered statistically significant when the initial *p* value was < 0.001 and the corrected *p* value, incorporating NBS correction following 5000 permutations, was < 0.05 for multiple comparisons.

## 4 Results

### 4.1 Statistical Results of Clinical Data and Cognitive Function of CKD Patients

This study investigates the prevalence rates of hypertension, diabetes, and dyslipidemia among NDD‐CKD and DD‐CKD patients, and assesses the concentrations of hemoglobin, red blood cells, albumin, urea nitrogen, creatinine, and phosphate levels. The statistical analysis reveals significant differences in the total MMSE and MoCA scores between the two CKD groups and the Normal Control (NC) group, with a *p* value < 0.001. Notably, significant disparities in protein and calcium levels were observed in NDD‐CKD patients compared to the NC group, with a *p* value < 0.05. Furthermore, significant differences were found in protein, creatinine, calcium levels, and MES and MoCA total scores between NDD‐CKD and DD‐CKD patients, with a *p* value < 0.001. In addition, phosphate levels in NDD‐CKD patients were statistically significant when compared to DD‐CKD patients, at *p* < 0.05. The detailed findings are presented in Tables 1, 2, 3.

**TABLE 1:** Demographic of the CKD and NC groups.

	CKD (*N* = 64)		NC (*N* = 43)	*T*/*X* ^2^ value			*p* value		
	NDD‐CKD group (*N* = 26)	DD‐CKD group (*N* = 38)		A	B	C	A	B	C
Age (years)	56.88 ± 11.70	58.06 ± 11.07	55.64 ± 9.70	0.47	−0.99	−0.39	0.64	0.32	0.69
Male (%)	17 (65.38)	18 (58.06)	28 (66.67)	0.01	0.57	0.32	0.91	0.45	0.57
Duration of disease (years)	3.00 ± 2.99	5.00 ± 4.50	NA	NA	NA	−1.93	NA	NA	0.05
Hypertension (%)	23 (88.46)	28 (90.32)	5 (11.90)	38.86	44.28	0.05	0.00^**^	0.00^**^	0.82
Diabetes (%)	19 (73.08)	18 (58.06)	1 (2.38)	38.65	28.72	1.40	0.00^**^	0.00^**^	0.24
Dyslipidemia (%)	17 (65.38)	23 (74.19)	2 (4.76)	29.31	38.18	0.52	0.00^**^	0.00^**^	0.47

*Note*: A—Comparison between the NC and NDD‐CKD groups; B—comparison between the NC and DD‐CKD groups; C—comparison between the NDD‐CKD and DD‐CKD groups.

^*^ *p* < 0.05.

^**^ *p* < 0.01.

**TABLE 2:** Clinical characteristics of the CKD and NC groups.

	CKD (*N* = 64)		NC (*N* = 43)	*T*/*X* ^2^ value			*p* value		
	NDD‐CKD group (*N* = 26)	DD‐CKD group (*N* = 38)		A	B	C	A	B	C
Hemoglobin (g/dL)	104.85 ± 23.66	112.61 ± 18.24	134.95 ± 16.27	−5.71	5.51	−1.40	0.00^**^	0.00^**^	0.17
Erythrocyte pressure (%)	31.85 ± 6.61	35.06 ± 5.78	40.45 ± 4.24	−5.92	4.60	−1.95	0.00^**^	0.00^**^	0.06
Protein (g/dL)	59.96 ± 9.55	67.42 ± 7.33	65.26 ± 5.50	−2.54	−1.44	−3.31	0.02^*^	0.16	0.002^**^
Albumin (g/dL)	32.50 ± 6.63	35.19 ± 4.62	38.24 ± 2.72	−4.20	3.27	−1.75	0.00^**^	0.002^**^	0.09
Urea nitrogen (mg/dL)	14.30 ± 11.47	18.96 ± 5.02	5.02 ± 1.36	4.11	−15.046	−1.920	0.000^**^	0.000^**^	0.064
Creatinine (mg/dL)	392.62 ± 328.65	969.35 ± 297.85	67.26 ± 16.41	5.04	−16.844	−6.946	0.000^**^	0.000^**^	0.000^**^
Calcium (mg/dL)	2.19 ± 0.20	2.37 ± 0.19	2.30 ± 0.11	−2.54	−1.71	−3.36	0.02^*^	0.09	0.001^**^
Phosphate (mg/dL)	1.45 ± 0.48	1.79 ± 0.73	1.09 ± 0.27	3.97	−5.06	−2.01	0.00^**^	0.00^**^	0.049^*^

*Note*: A—Comparison between the NC and NDD‐CKD groups; B—comparison between the NC and DD‐CKD groups; C—comparison between the NDD‐CKD and DD‐CKD groups.

^*^ *p* < 0.05.

^**^ *p* < 0.01.

**TABLE 3:** Cognitive scores of the CKD and NC groups.

	CKD (*N* = 64)		NC (*N* = 43)	*T*/*X* ^2^ value			*p* value		
	NDD‐CKD group (*N* = 26)	DD‐CKD group (*N* = 38)		A	B	C	A	B	C
Total MMSE score	27.00 ± 1.26	22.39 ± 2.75	28.38 ± 1.27	−4.37	11.27	8.34	0.00^**^	0.00^**^	0.00^**^
MoCA scale total score	25.46 ± 2.14	23.55 ± 2.28	27.29 ± 1.60	−3.75	7.83	3.25	0.001^**^	0.00^**^	0.002^**^

*Note*: A—Comparison between the NC and NDD‐CKD groups; B—comparison between the NC and DD‐CKD groups; C—comparison between the NDD‐CKD and DD‐CKD groups.

^*^ *p* < 0.05.

^**^ *p* < 0.01.

### 4.2 NBS Network Analysis in NDD‐CKD Patients

**FIGURE 1:**
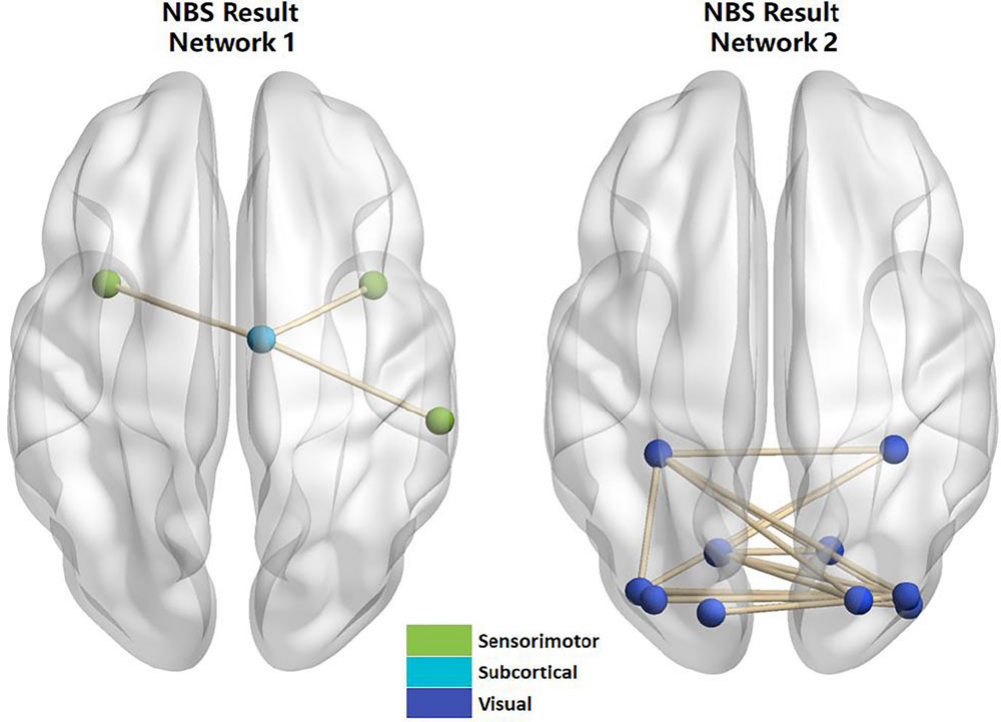
Functional results of network connectivity for NDD‐CKD patients.

The outcomes of the network‐based FC and network‐based integrative analysis are depicted in Figure 1 and summarized in Table 4. NDD‐CKD patients exhibited reduced FC within two distinct brain networks relative to the NC group, with statistical significance at *p* < 0.05. Notably, network 1 demonstrated significantly attenuated connectivity, which was associated with the right dorsolateral prefrontal cortex (DCG), right superior medial frontal gyrus (SMG), and bilateral insula (INS). Furthermore, network 2 exhibited a marked reduction in connectivity function and was correlated with regions including the bilateral lingual gyrus (LING), bilateral superior occipital gyrus (SOG), bilateral middle occipital gyrus (MOG), bilateral inferior occipital gyrus (IOG), and bilateral fusiform gyrus (FFG).

**TABLE 4:** Statistical results of NBS for people with NDD‐CKD and NC groups.

Network	Number of nodes	NBS *p* value	From	To	*T* value
1	4	0.0312	INS.L	DCG.R	3.60
			INS.	DCG.R	3.75
			DCG.R	SMG.R	3.70
2	10	0.0000	LING.	LING.R	3.59
			LING.	SOG.R	4.30
			SOG.	SOG.R	3.95
			LING.	MOG.R	4.26
			SOG.	MOG.R	3.59
			MOG.	MOG.R	3.96
			MOG.R	IOG.	4.36
			MOG.R	IOG.R	4.05
			IOG.	IOG.R	5.32
			SOG.R	FFG.L	3.50
			MOG.R	FFG.L	4.23
			IOG.	FFG.L	3.65
			IOG.R	FFG.L	5.10
			LING.	FFG.R	4.26
			IOG.	FFG.R	3.80
			FFG.L	FFG.R	4.14

### 4.3 NBS Network Analysis in DD‐CKD Patients

**FIGURE 2:**
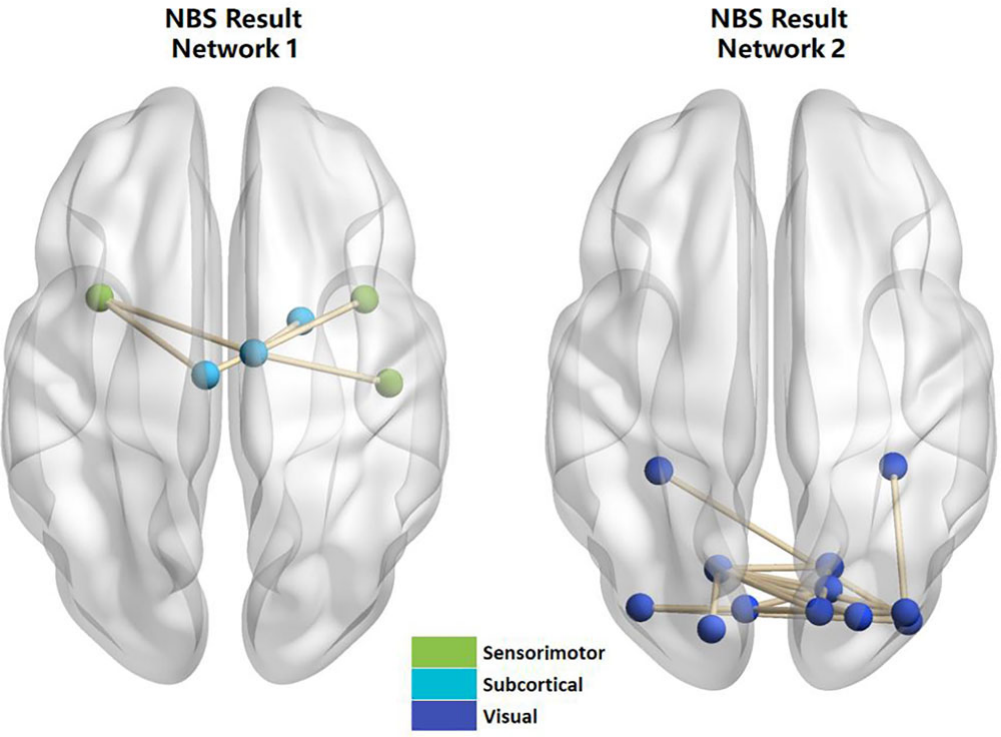
NBS network function displayed in DD‐CKD patients.

The results of the NBS network analysis are presented in Figure 2 and tabulated in Table 5. DD‐CKD patients exhibited reduced functional brain connectivity within two distinct networks, with statistical significance at *p* < 0.05. Changes in FC within network 1 were associated with bilateral DCG, right parietal association lobe (PAL), right Heschl's gyrus (HES), and bilateral INS. For network 2, FC changes were correlated with bilateral cingulate association lobe (CAL) perisylvian cortex, right cuneus (CUN), bilateral LING, bilateral SOG, right MOG, bilateral IOG, and bilateral FFG. The discrepancies observed were also statistically significant when compared to the NDD group.

**TABLE 5:** Results of NBS in patients with DD‐CKD compared with the NC group.

Network	Number of nodes	NBS *p* value	From	To	*T* value
1	6	0.0054	INS.L	DCG.L	4.38
			INS.	DCG.L	3.75
			INS.	DCG.R	3.78
			INS.	DCG.R	3.6
			DCG.R	PAL.R	3.58
			DCG.R	HES.R	3.61
2	12	0.0006	CAL.	CAL.R	4.5
			CAL.	CUN.R	3.61
			CAL.R	LING.	4.23
			CUN.R	LING.	4.47
			CUN.R	LING.R	4.01
			LING.	LING.R	3.8
			LING.	SOG.	3.96
			LING.	SOG.R	4.08
			LING.	MOG.R	3.58
			MOG.R	IOG.	3.56
			MOG.R	IOG.R	3.69
			IOG.	IOG.R	3.72
			IOG.R	FFG.L	3.55
			MOG.R	FFG.R	3.79

### 4.4 Correlation Between Brain Function Connectivity and Kidney Function

**FIGURE 3:**
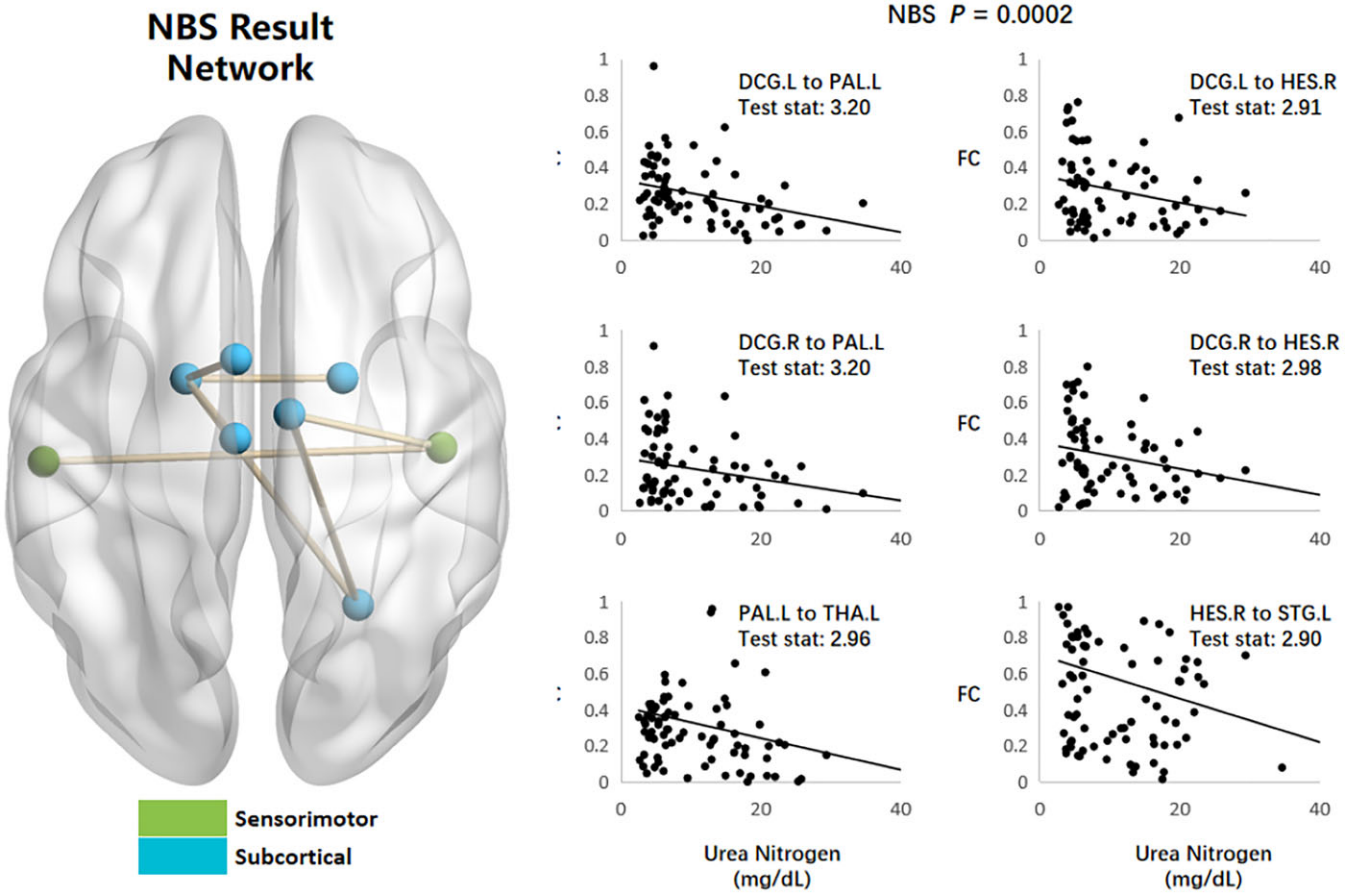
Correlation between brain function connectivity and kidney function.

The relationship between brain FC and renal function is illustrated in Figure 3. Brain FC was found to be inversely correlated with renal function and serum urea nitrogen levels. The brain FC network demonstrated associations with bilateral DCG, left PAL, left temporal‐hippocampal area, and right PAL.

## 5 Discussion

Cognitive dysfunction is a prevalent condition among patients with CKD, with incidence rates varying depending on the stage of renal impairment. Approximately 20%–50% of patients in the medium stage of CKD exhibit cognitive impairment, while this figure can rise to as high as 24%–67% among those receiving renal replacement therapy (Conrin et al. 2018). Advanced imaging techniques, including fMRI and diffusion tensor imaging, have been instrumental in elucidating the functional and structural dynamics of the temporoparietal occipital area, which is crucial for accurate disease diagnosis and tailored treatment strategies. Although significant differences in total MMSE and MoCA scores were observed between the DD‐CKD and NDD‐CKD groups, no significant differences in brain tissue network connections were found. This result can be attributed to the different stages of cognitive impairment and renal function deterioration within these groups. The DD‐CKD group, being dialysis‐dependent, likely experiences more severe cognitive decline, as reflected in the lower cognitive scores, despite similar FC patterns in the brain networks.

In the present investigation, cognitive function assessments were conducted on selected participants, revealing significant discrepancies between CKD patients and healthy controls. Specifically, CKD patients exhibited markedly lower cognitive function scores compared to their healthy counterparts. Furthermore, within the CKD patient group, individuals with DD‐CKD exhibited significantly lower scores in both MMSE and MoCA assessments compared to those with NDD‐CKD. These findings collectively indicate that cognitive impairment is prevalent among CKD patients, varying in severity.

Although qPCR analysis did not reveal significant upregulation of SLC1A2 or IL‐6 transcripts, this absence of transcriptional change does not contradict the imaging findings. It is important to note that peripheral blood measures may not fully capture brain‐specific molecular alterations, and functional impairment can occur despite stable mRNA levels. Indeed, recent CKD models have shown astrocytic dysfunction and gap‐junction uncoupling in the setting of unchanged transcript abundance (Bedner and Steinhäuser 2023). One plausible explanation is post‐transcriptional regulation—for instance, miR‐29‐mediated suppression of EAAT2 translation—which could reduce glutamate clearance and thereby contribute to the frontoparietal disconnectivity observed in our fMRI analyses (Dalgaard et al. 2022). In this way, the molecular and imaging findings may be viewed as complementary rather than contradictory.

Further analysis using fMRI technology revealed that brain functional connections in both NDD‐CKD and DD‐CKD patients were negatively correlated with renal function, aligning with previous research outcomes. Studies have highlighted that individuals with cognitive dysfunction often exhibit altered functional connections, which may be indicative of underlying neural abnormalities (Abdelnour et al. 2018). In patients with ESRD, imbalances in the segregation and integration of brain subnetworks have been suggested, although the precise mechanisms remain elusive. For example, a study reported significant reductions in global efficiency, local efficiency, clustering coefficient, and small‐worldness index in ESRD patients, indicating disrupted integration and segregation within brain networks, which were closely associated with cognitive performance (Park et al. 2020). In addition, this study found that decreased structural and FC efficiency was significantly correlated with lower MoCA scores (Park et al. 2020). More notably, a recent dynamic fMRI study (Li et al. 2022) revealed reduced temporal flexibility of functional networks in ESRD patients, including fewer state transitions and lower variance in efficiency measures, suggesting impaired dynamic reconfiguration capabilities of brain networks, which were linked to deficits in executive function and attention.

Compared with NCs, patients with NDD‐CKD exhibited reduced FC primarily within the frontal‐insular and visual networks. Specifically, disruptions were noted in the right DCG, SMG, and bilateral INS, as well as in bilateral LING, SOG, MOG, IOG, and FFG. These regions are associated with attention control, sensory integration, and visuospatial processing, suggesting early and localized network dysfunction. In contrast, DD‐CKD patients demonstrated more widespread network alterations involving bilateral DCG, INS, right PAL, and HES, along with additional impairment in the visual‐cingulate pathway, including bilateral CAL and right CUN. Several of these regions, notably DCG and PAL, also showed negative correlations with renal function indices. Given the significantly lower MMSE and MoCA scores in the DD‐CKD group, these findings support a pattern of progressive network disintegration linked to both declining kidney function and worsening cognitive performance. These results were consistent with prior fMRI studies in ESRD populations, which had also shown disrupted connectivity in large‐scale brain networks. A study reported reduced FC within core default mode network hubs—specifically the posterior cingulate cortex, precuneus, and medial prefrontal cortex—with such disruptions correlating with hemoglobin levels and estimated GFR (Ni et al. 2014). More recently, a meta‐analysis of rs‐fMRI studies confirmed consistent alterations in major functional networks in ESRD patients, including the default mode, sensorimotor, and executive control networks (Song et al. 2023). The impaired integration of these distributed networks was believed to represent a potential neural basis for cognitive and affective disturbances associated with advanced kidney disease.

Although our fMRI approach cannot resolve receptor‐level distributions, prior postmortem work in ESRD patients has suggested higher glucocorticoid receptor (GR) density within the dorsal ACC but not in occipital cortices (He et al. 2025). Such regional gradients could partly account for the preferential vulnerability of frontoparietal networks to uremic stress, though this remains speculative and requires further validation. Translational validation could leverage human‐induced pluripotent stem cell‐derived astrocytes exposed to uremic serum, enabling causal testing of GR‐blockade on network excitability (Yang et al. 2022).

Regional vulnerability might be related to differential GR expression. Postmortem work has suggested higher GR density in the DCG of CKD patients compared with occipital regions (Yu et al. 2023), which could help explain the more pronounced FC reductions we observed in frontoparietal networks. However, this interpretation remains tentative and warrants further confirmation in future studies. Moreover, advancements in fMRI‐based machine learning approaches have provided valuable insights into the diagnosis of neuronal diseases (Yang et al. 2023; Zhou et al. 2024; Xiao et al. 2024). The clinical utility of fMRI technology in studying brain connection function has been widely acknowledged (Li et al. 2017; Li et al. 2023; Zhou et al. 2024). The application of fMRI to identify reduced cognitive function in ESRD patients not only underscores the impairment of network connection strength but also offers a potential avenue for early detection and intervention in neurocognitive disorders associated with renal impairment.

Finally, emerging evidence for neuro‐glio‐vascular coupling suggests that astrocytic endfoot AQP4 polarization is compromised in CKD (Battaglia et al. 2025; Battaglia et al. 2024). Integrating arterial‐spin‐labeling perfusion MRI with the present rs‐fMRI protocol could disentangle vascular from neuronal contributions to the observed disconnectivity. In addition, uremia‐induced impairment of astrocytic endfoot AQP4 polarization—recently reported in rodent CKD models (Cheng et al. 2013), may underlie the vascular contribution to the observed functional disconnectivity.

This study has limitations. While the NBS method is appropriate for identifying network‐level differences, effect sizes for correlations between connectivity and clinical measures were not assessed in this study, limiting the interpretability of brain–kidney associations. Future research should incorporate standardized effect size metrics to improve clinical relevance.

## 6 Conclusion

This study demonstrates that CKD patients exhibit impaired brain FC and cognitive performance, with DD‐CKD patients showing more extensive network disruptions compared to those with NDD‐CKD. These alterations were associated with worse renal function and lower MMSE and MoCA scores. rs‐fMRI may serve as a valuable tool for the early detection of neurocognitive decline in CKD.

## Author Contributions

Ying Liu was responsible for the conceptualization of the study. The methodology was developed by Ying Liu, Yingying Wang, and Huan Yu. Liling Peng conducted the investigation and validation. Ning Wu created the visualizations. Resources were provided by Ning Wu, Chunhua Song, and Chaoyang Zhang. Ying Liu and Yan Cai drafted the original manuscript, while Zhenwei Wang, Yiqing Sun, and Xin Gao were involved in the review and editing process. Project administration was managed by Ning Wu, Chunhua Song, and Chaoyang Zhang. Xin Gao served as the corresponding author for the manuscript.

## Ethics Statement

The experimental protocols were approved by the Ethics Committee of Liangxiang Teaching Hospital of Capital Medical University and carried out in accordance with the Helsinki Declaration, with ethics number 2016174.

## Conflicts of Interest

The authors declare no conflicts of interest.

## Peer Review

The peer review history for this article is available at https://publons.com/publon/10.1002/brb3.70947.

## Data Availability

Data are available on request from the corresponding author.
